# Legislation for Midwives

**Published:** 1900-03-24

**Authors:** 


### The Institutional Workshop.

### LEGISLATION FOR MIDWIVES.

From a Correspondent.

### I.—The Need for Reform.

A movement in favour of regulating the professional work of the midwife has now been in progress some years, and has manifested itself in all the usual methods open to the advocates of reform in this country. An association has been formed for promoting the compulsory registration of midwives. Bills have been introduced by private members session after session ever since 1890 dealing with the question, and a Select Committee of the House of Commons so far back as 1893 examined witnesses, heard the opinion of experts, and declared in their report that legislation was imperatively required. In July last a deputation of those in favour of legislation on this subject was received by the Duke of Devonshire at the Privy Council Office; and the Lord President of the Council then stated that, although he was unable to give much hope of the Government undertaking this measure next session, he considered the need for legislation had been conclusively established.

So much, then, has been accomplished, and now a Bill has passed its second reading. The Royal Colleges of Physicians and Surgeons, the General Medical Council, the societies representing the better class of midwives, the Executive Committee of the County Councils' Association, the Coroners' Society of England (more deeply concerned than may at first sight appear), and, lastly, Her Majesty's Privy Council are all of one mind concerning the undesirability of leaving the midwives in their present irresponsible position.

Between agreeing that something ought to be done and agreeing upon the best thing to do, a wide gulf is fixed. Notwithstanding all the energy expended during the last ten years in the attempt to bridge this gulf there still exists a respectable body of opposition to the scheme as at present formulated, and some of those who most clearly recognise existing evils are still counselling deliberation and delay.

The condition of affairs which has occasioned this extensive movement in favour of the State direction of the midwives' calling is nothing new and can be briefly explained. Statistics collected with great care by the Obstetrical Society of London show that in about 62 per cent. of the births of the kingdom the mother is attended not by a medical practitioner but by a midwife. The proportion is often only 5 to 10 per cent. in towns, but varies between 30 and 90 per cent. in country districts. It has been estimated that about 10,000 women in England exercise the calling of a midwife with more or less regularity. These women are under no supervision; they need no licence to practice, and, as a direct consequence, they need, and often possess, for the exercise of their calling no further knowledge than they have picked up by casual observation. The majority of these women are careful, honest, and, after their lights, skilful enough. But doctors all over the country (and here the coroners' evidence is very valuable) are at one in their strong conviction of the ignorance, incompetence, and malpraxis of many midwives whom they are entirely powerless to restrain from doing more mischief. They see daily evidence that the lives of mothers are endangered for want of the most elementary notions of cleanliness, and that a woman's subsequent health is often wrecked by ill-judged interference or want of ordinary skill on the part of the midwife. Worse than all, it appears that from 30 to 40 per cent. of the blind owe their calamity to infantile ophthalmia, the direct result of the nurse's dirt and carelessness at their birth.

All this is transparently bad. We do not require to reckon up the number of women who perished last year from puerperal fever or who were treated in hospitals for injuries ignorantly inflicted by their nurses, to be convinced of the fact that the duties of a midwife are too responsible to be left in the hands of a body of women accountable to none.

As a matter of fact the licensing of midwives has only during the last 150 years fallen entirely into disuse in England. In mediæval times, when the intervention of either physician or surgeon at a birth would have been regarded as the violation of a woman's honour, the midwives were a highly important class. They took a solemn and very complicated oath before the bishop of their diocese as a preliminary to practice, and received a licence from him which conferred on them, among other powers, authority to baptise infants who were in danger of dying before the priest could be fetched. The bishops' share in the licensing of midwives ceased after the Hampton Court Conference in 1603, at which lay baptism, on account of many abuses, was disallowed. But midwives continued to receive licences, for which they paid about eighteen shillings, as late as into the eighteenth century. In order to procure a licence they had to show recommendations from their parish priest, and from matrons whom they had delivered; but as no qualification except Protestantism was implied by the possession of these licences, they came to be held in light esteem, and not being since the Reformation legally necessary, they fell gradually into entire disuse.

The licensing of midwives is then so far from being a novelty that it is only in comparatively modern times that no system for their regulation has been in force. The midwives' calling, however, has been in very ill-repute for several hundred years, and there can be no doubt that, long ere this, some one of the many measures which have been proposed during this period for its more effectual organisation would have been adopted had it not been for the rise of an entirely new feature in the practice of obstetrics. This was no less than the intervention of various eminent physicians in the midwives' calling. In 1540 Thomas Raynold issued the first book on the subject ever published in England, entitled "The Birthe of Mankynde," being a translation from Eucharius Rhodion, a German author. A famous treatise of Guillemeau on "Childbirth; or, the Happie Deliverie of Women," was shortly after translated into English and ran through many editions. These and other translated works appear to have roused English physicians to the study of obstetrics. Almost the first to lend his attention to this subject was

William Harvey, and from his time onward isolated members of the medical profession, notably the Chamberlens, father and sons, devoted their energies to raising this to a serious branch of the profession. It was long, however, before obstetrical work assumed any dignity in the eyes of the medical practitioner, and it was hardly till this century that it became an essential feature of medical education. The man-midwife, as he was long awkwardly called, was for many years a being of status inferior to either physician and surgeon, and kept himself entirely to his own department. Very great ladies employed the man-midwife under the direction of their own physician, who prescribed the inevitable drugs while his subordinate carried out his orders. It grew to be thought the correct thing to employ these medical midwives, and those ladies who still clung to the old prejudice in favour of their own sex began to think it necessary to have the doctor "waiting in the next room." The perfecting and common use of forceps gave the final blow to female supremacy in this line, for the use of surgical instruments could be permitted to none but surgeons.

*(To be continued.)*

